# Supplementary material for: A Global Screen for Assembly State Changes of the Mitotic Proteome by SEC-SWATH-MS
Source: Cell Syst. 2020 Feb 26;10(2):133–155.e6. doi: 10.1016/j.cels.2020.01.001 (PMC7042714; doi:10.1016/j.cels.2020.01.001)

Q08623 | HDHD1_HUMAN | PUDP DXF68S1E FAM16AX GS1 HDHD1 HDHD1A
Monomer MW [kDa]: 25.249 Monomer expected elution fraction: 52

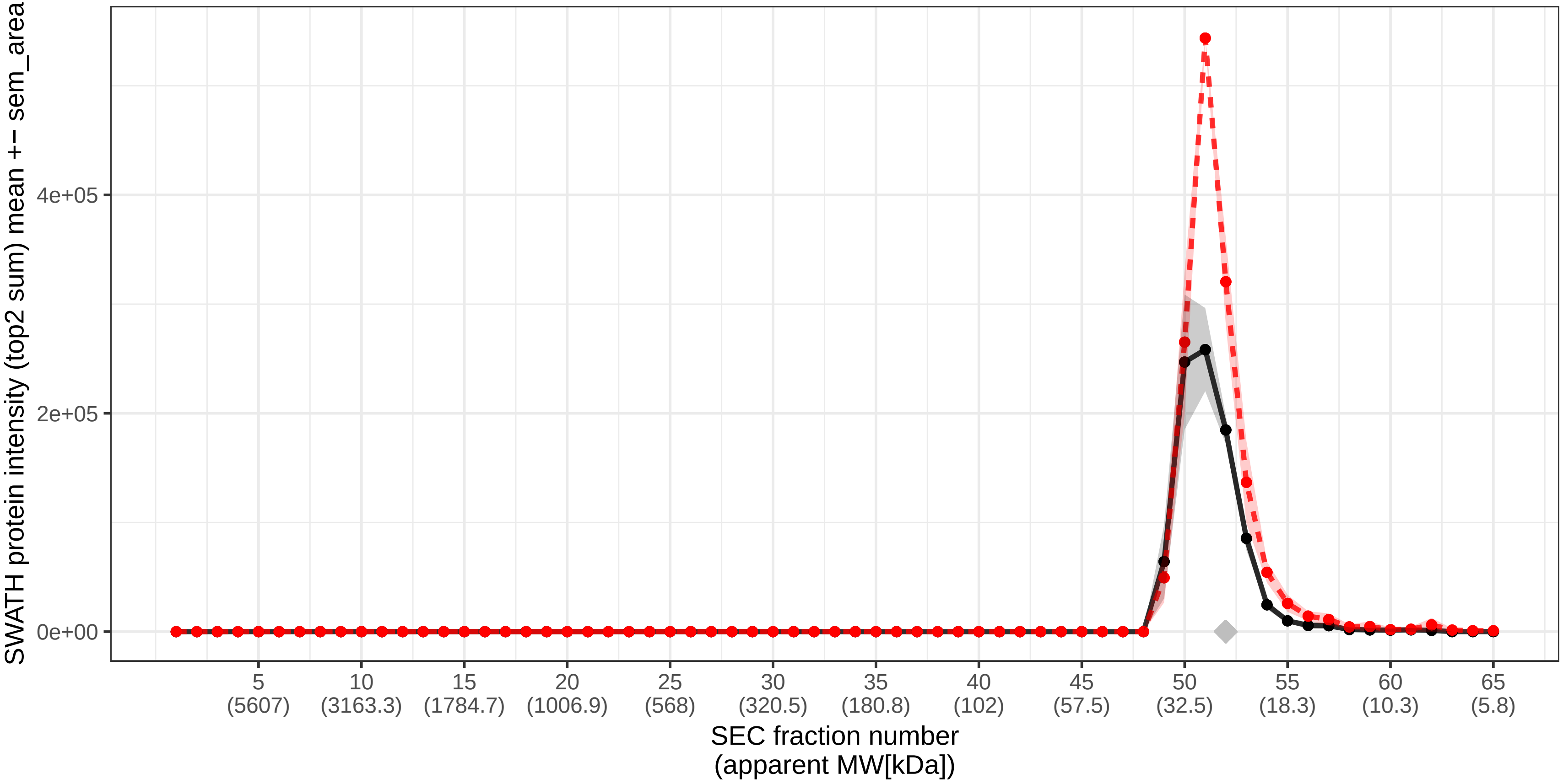

Supplement: Data S1. SEC-SWATH-MS Protein Chromatograms, Related to Figure 1 [file mmc6.zip › SECchrom_Q08623_HDHD1_HUMAN_PUDP_DXF68S1E_FAM16AX_GS1_HDHD1.pdf]
